# Projections of maternal mortality ratios in Bangladesh

**DOI:** 10.7189/jogh.14.04015

**Published:** 2024-01-26

**Authors:** Etsuko Nishimura, Daisuke Yoneoka, Md. Obaidur Rahman, Yuki Yonekura, Yaeko Kataoka, Erika Ota

**Affiliations:** 1 Graduate School of Nursing Science, St. Luke’s International University, Tokyo, Japan; 2 Center for Surveillance, Immunization, and Epidemiologic Research, National Institute of Infectious Diseases, Tokyo, Japan; 3 Tokyo Foundation for Policy Research, Tokyo, Japan; 4 Center for Evidence-Based Medicine and Clinical Research, Dhaka, Bangladesh

## Abstract

**Background:**

The objective of this study was to predict when Bangladesh would achieve Sustainable Development Goal Target 3.1, which is to reduce the maternal mortality ratio (MMR) to less than 70 per 100 000 live births.

**Methods:**

We used secondary data from the 1993 to 2017 Bangladesh Demographic and Health Surveys and other sources to project the MMR until 2060 under several scenario assumptions using an autoregressive moving average model with exogenous variables (ARMAX). Explanatory variables were selected based on the three delays model, and a reference forecast and four practical scenarios were simulated: Scenario 1 assumed a 4% annual increase in institutional deliveries, Scenario 2 followed the national goals, the reference forecast and Scenario 3 varied in terms of district-wise increase rates (Scenario 3 had a lower rate of increase), and Scenario 4 assumed minimal changes in institutional deliveries.

**Results:**

Scenario 1 was the earliest, with an MMR of <70 per 100 000 live births in 2026. Scenario 2 would meet the target of <70 per 100 000 live births in 2029. The reference forecast had the third lowest MMR, with 69.78 per 100 000 live births (95% prediction intervals (PI) = 32.44 to 107.11) in 2049. Although the MMR for Scenario 3 decreased slowly, it would not reduce below 70 per 100 000 live births by 2060. Scenario 4, which had the highest MMR, also resulted in the MMR not reducing below 70 per 100 000 live births by 2060.

**Conclusions:**

To increase the institutional delivery rate and reduce the MMR, as in Scenarios 1 and 2, it is necessary to improve the institutional delivery rate in regions with low institutional delivery rates. Additionally, health facilities need to provide appropriate quality medical care to increase the institutional delivery rate and contribute to a decrease in the MMR, as shown by the results of this study.

The global maternal mortality ratio (MMR) has decreased from 342 per 100 000 live births in 2000 to 211 per 100 000 live births in 2017 [1]. However, 94% of maternal deaths occur in low- and middle-income countries (LMICs), and the MMR remains high in some LMICs [2]. The causes of maternal death have been categorised into direct and indirect maternal causes [2]. Approximately 73% of all maternal deaths worldwide occur because of direct obstetric causes, such as severe bleeding, hypertension, and infectious diseases [3]. If women have access to appropriate health care services during labor, delivery, and the 48 hours after delivery, these causes can be prevented [4,5]. On the other hand, the indirect causes of maternal mortality have been categorised as medical disorders, including HIV-related maternal mortality, poor mental health, and other indirect causes [3,6].

Target 3.1 of the Sustainable Development Goals (SDGs) aims to reduce the MMR [7]. More specifically, it aims to ‘by 2030 reduce the global maternal mortality ratio to less than 70 per 100 000 live births’ [7]. This is measured by Indicators 3.1.1 and 3.1.2. Indicator 3.1.1 is the MMR, while the other indicator is defined as the ‘proportion of births attended by skilled health personnel’ [7]. Although the proportion of births assisted by skilled health care workers has increased globally, a wide gap exists among regions, particularly in LMICs.

Bangladesh, a country in Southern Asia, is one of the countries where the MMR has declined dramatically from 434 per 100 000 live births in 2000 to 173 per 100 000 live births in 2017 [1]. However, its MMR was higher than the average MMR in Southern Asia (157 per 100 000 live births in 2017) [1]. The proportion of deliveries attended by skilled health personnel was 53% in 2017 [8]. According to the 2017–2018 survey, these health workers assisted in childbirth primarily at hospitals or clinics, and the majority of births that occurred at home were assisted by nonhealth care workers [8].

According to the National Strategy for Maternal Health 2019–2030, the government intends to increase the percentage of births assisted by skilled health personnel to 60% in 2020, 80% in 2025, and 90% in 2030 and reduce the MMR for the same years to 145 per 100 000 live births, 100 per 100 000 live births, and 70 per 100 000 live births, respectively [9]. However, one study projected Bangladesh's MMR in 2030 to be between 150 and 200 per 100 000 live births [10], and this range indicates that target 3.1 of the SDGs will not be achieved.

The objective of this study was to predict when Bangladesh would achieve Target 3.1, namely, to reduce the MMR to less than 70 per 100 000 live births.

## METHODS

### Overall structure of forecasting model for the maternal mortality ratio

To predict an MMR of less than 70 per 100 000 live births in Bangladesh, the available MMR data from 1993 to 2017 were used for modeling. The variables used to predict the MMR were determined based on the three delays model [11], considering the following phases, namely, Phases 1–3. In each phase of the three delays model, the factors affecting maternal mortality were defined. Phase 1 was defined as socioeconomic or cultural factors, Phase 2, as accessibility of health facilities, and Phase 3, as quality of care [11]. The study was reported in accordance with the Guidelines for Accurate and Transparent Health Estimates Reporting (GATHER) statement (Table S1 in the **Online Supplementary Document**) [12].

### Data sources

Based on these three factors, the available data were collected from the six data sources listed below.

#### Demographic health survey

Data from the DHS programme, including data from a population-based household survey conducted from 1993 to 2017, were used. These representative data included respondent characteristics, household characteristics, and information regarding sexual and reproductive health, women's empowerment, neonatal and child health, HIV, nutrition, access to health care facilities, and availability of health services. In more than 90 countries, trained personnel collected these representative data using a standardised questionnaire [13]. The survey is usually conducted every five years to allow for comparisons over time. In Bangladesh, the survey is conducted approximately every three to four years.

#### Socio-demographic index

The socio-demographic index (SDI) is the geometric mean of three factors: (a) the total fertility rate under the age of 25 years, (b) income per capita, and (c) average educational attainment of the population aged 15 years or older. The SDI scores are scaled from 0 to 1, with 0 indicating the highest fertility, lowest income, and fewest years of schooling and 1 indicating the lowest fertility rate, highest income, and most years of schooling [14].

#### World Bank open data

The World Bank supports many programmes for collecting cross-border data and creates international data sets based on the data generated by national statistical systems [15].

#### Data from a World Health Organization document

The document ‘Trends in Maternal Mortality: 1990 to 2015’ describes the number of maternal deaths, MMR, and lifetime risk in each country, region, and the world [16]. The MMR data from this document were used for modeling.

#### Multiple indicator cluster surveys

The Multiple Indicator Cluster Surveys (MICS) programme was designed to be representative at the national level and to collect data on children and women. The surveys have been conducted in 118 countries, including Bangladesh [17].

#### Data from a Bangladesh government document

Data from the ‘Bangladesh National Strategy for Maternal Health 2019–2030’, compiled by the Ministry of Health and Family Welfare (2019) [9] were used to create a scenario.

### Variables in prediction model

The appropriate variables representing the three factors defined above were selected based on discussions with experts familiar with maternal and child health care in Bangladesh: ‘Socio-Demographic Index (SDI)’ for socioeconomic or cultural factors, ‘percentage of sample clusters with a health facility within 5 km’ for accessibility to health facilities, and ‘percentage of women receiving quality antenatal care (ANC)’ and ‘facility delivery rate’ for quality of care. Variables were selected from national-level data and were chosen to be measured more frequently between 1993 and 2017.

### Interpolation for time series data

The data source for MMR was primarily the World Bank open data repository, and the data source for the SDI was the 2019 data from the Global Burden of Disease (GBD). The DHS data were mainly used for the other variables. Because the DHS is usually conducted in Bangladesh once every three to four years, the yearly data were interpolated. Spline interpolation was used to interpolate missing values for variables, except for those related to ANC quality. For variables related to the quality of ANC, linear imputation was used because of the small number of internal time points.

### Scenarios for facility delivery rate

A reference forecast and four alternative scenarios (best, moderate 1, moderate 2, and worst) were simulated by changing the future value of one variable during the 2018–2060 period, for which the observed values of the MMR were unavailable. Creating these scenarios made it possible to predict changes in the MMR for various patterns. The variable used in the scenarios was facility delivery rate, as it has a more direct impact on maternal mortality than the other variables.

#### Reference forecast scenario

In the reference forecast, regional disparities were reflected in the scenario. Based on the institutional delivery rates, by division, of the 2017 DHS, the three divisions of Barishal, Mymensingh, and Sylhet, with less than 40%, were considered ‘low divisions’; the two divisions of Chattogram and Rangpur, with around 45%, were considered ‘moderate divisions’; and the three divisions of Rajshahi, Dhaka, and Khulna, with more than 50%, were designated as ‘high divisions. The institutional delivery rate in the ‘low divisions’ was assumed to increase by 1.25% per year. The ‘moderate divisions’ were assumed to have a 3.0% annual increase in institutional delivery rates, but this increase was expected to reduce to 1% when the rate reached 75 and 0.5% when it reached 85%. The reason for the drop in the annual increase rate when the institutional delivery rate reaches 75% is that when the institutional delivery rate in neighboring India exceeded 75%, the annual increase rate decreased from an average of 5.0% (between 2005 and 2013) to an average of 0.1% (between 2014 and 2016) [18]. Since the institutional delivery rate in Bangladesh increased by 4% per year, the highest on record, between 2014 and 2017, the scenario for the ‘high divisions’ assumed a 4% annual increase in the institutional delivery rate. When the institutional delivery rate reached 75%, the annual increase was assumed to be 2.0%, and when it reached 85%, the annual increase was assumed to be 1.0%. When the institutional delivery rate reached 90%, the annual increase was assumed to be 0.5%, and when it reached 95% and 97%, the annual increase was assumed to be 0.25% and 0.12%, respectively. Based on the above hypotheses, the institutional delivery rate for each of the eight districts was determined on a yearly basis and the average rate of increase for each year was calculated. This value was used as the institutional delivery rate for the scenarios.

#### Four alternative scenarios

Scenario 1 assumed that this 4% growth would continue through 2018 and beyond, without considering regional differences. It assumed a 2% annual increase when the facility delivery rate reached 75%, a 1% annual increase when it reached 85%, and a 0.5% annual increase when it reached 90%. When the institutional delivery rate reached 95%, the annual rate of increase was assumed to be 0.25%. Scenario 2 assumed that the institutional delivery rate would increase to 85% by 2030, based on the targets of the National Strategy for Maternal Health 2019–2030 [9]. Scenario 3 also reflected regional disparities. The institutional delivery rate in the ‘low divisions’ was assumed to increase by 0.5% per year. The ‘moderate divisions’ were assumed to have a 1% annual increase in the institutional delivery rate, but this increase was expected to drop to 0.5% when the rate reached 75%. The ‘high divisions’ were assumed to have a 4% annual increase in the institutional delivery rate, but it was expected to drop to 2% when the rate reached 75% and 1% when the rate reached 85%. Scenario 4 was the worst among the four scenarios. In the ‘low’ and ‘medium’ divisions, institutional delivery rates were assumed to barely change from the 2017 rate.

### Model for forecasting maternal mortality ratio

An autoregressive moving average model with exogenous variables (ARMAX) was used to forecast MMR. The ARMA model is the combination of an autoregressive (AR) model and a moving average (MA) model, and it is used in time-series analysis to represent stationary time series [19]. The ARMAX model can capture the time dependency between the present and previous time points, thus enabling us to obtain stable future predictions. When the order of the AR model is *p* and that of the MA model is *q*, the ARMA model is referred to as ARMA (*p*, *q*).

In this study, a model was selected to determine the order of *p* and *q*, and Akaike's information criterion (AIC) was computed to select the optimal models. Additionally, the Dickey–Fuller test was performed to examine the stationarity of the collected data. If non-stationarity is confirmed, it is possible to transform the data into a stationary process by taking the difference with *d*-order [19]. An ARMA (*p*, *q*) model with *d*-order differences is called an Auto Regressive Integrated Moving Average (ARIMA) (*p*, *d*, *q*) model [19].

In this study, the ARMAX model was used for simplicity; the AR and MA terms explained the effect of serial correlation, while the explanatory variables X included SDI, percentage of sample clusters with a health facility within 5 km, percentage of women receiving quality ANC, and facility delivery rate. The equation used in the analysis is as follows:



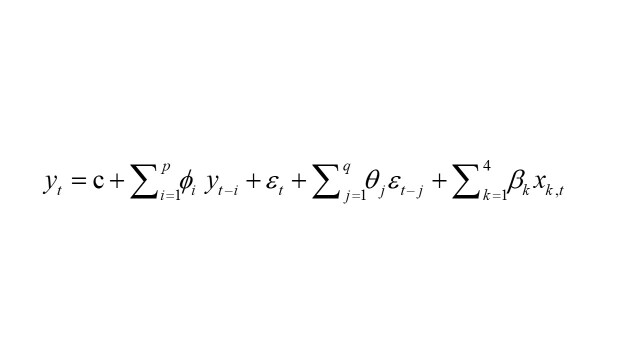



Here, *y_t_* is the outcome of interest at time t, *ϕ_i_*, *θ_j_*, and *β_k_* are the parameters of interest, and *ε_t_* is a white noise that is identically and independently distributed. Once the parameters are estimated, we can predict the future state and data given the estimated parameter set. Future MMR values and the corresponding 95% prediction intervals (PI) were calculated. The goodness of fit of the forecasting model was verified by fitting the actual observed values to the forecasting model after making predictions regarding the MMR. All analyses were conducted using R version 4.2.0.

### Ethical consideration

Secondary data were used in this study. Therefore, no ethical approval was required to conduct this study.

## RESULTS

The summarised data used to predict the MMR from 1993 to 2017 are presented in **Table 1**. The MMR was mainly based on the World Bank open data, and the SDI was based on the 2019 data from the Global Burden of Disease (GBD). The other variables were primarily obtained from DHS data. Because the DHS is usually conducted every three to four years in Bangladesh, data that could not be obtained were represented as not applicable. Figures S1–S4 in the **Online Supplementary Document** show the missing values for each variable, interpolated using spline interpolation or linear imputation.

**Table 1:** Maternal mortality ratio, SDI, accessibility to health facility, and utilisation of quality ANC and facility delivery

	World Bank open data	GBD 2019 data	DHS	DHS	DHS
**Year**	**MMR /100 000 live births**	**SDI**	**Percentage of sample clusters with health facility within 5 km (%)**	**Percentage of women receiving quality of ANC (%)**	**Facility delivery rate (%)**
1993	452*	0.290	89.9	na	3.5
1994	459*	0.297	na	na	na
1995	479†	0.304	na	na	na
1996	na	0.311	79.6	na	4.7
1997	na	0.317	na	na	na
1998	na	0.324	na	na	na
1999	na	0.330	na	4.0	8.7
2000	434	0.336	na	na	na
2001	423	0.343	na	na	na
2002	410	0.349	na	na	na
2003	395	0.355	na	na	na
2004	372	0.362	99.7	7.2	11.7
2005	343	0.369	na	na	na
2006	315	0.376	na	na	16.0‡
2007	297	0.384	98.0	7.2	17.2
2008	280	0.392	na	na	na
2009	269	0.400	na	na	na
2010	258	0.408	na	na	na
2011	248	0.416	99.6	na	28.8
2012	238	0.425	na	na	31.0‡
2013	227	0.433	na	na	na
2014	214	0.441	100	10.7	37.4
2015	200	0.449	na	na	na
2016	186	0.457	na	na	na
2017	173	0.466	99.7	17.7	49.4

ANC – antenatal care, DHS – demographic and health surveys, GBD – global burden of disease, MMR – maternal mortality ratio, na – not applicable, SDI – socio-demographic index

*Data from Bangladesh Bureau of Statistics and United Nations Children's Fund (UNICEF) (1996).

†Data from the World Health Organization (2015).

‡Data from Multiple Indicator Cluster Surveys.

**Figure 1:**
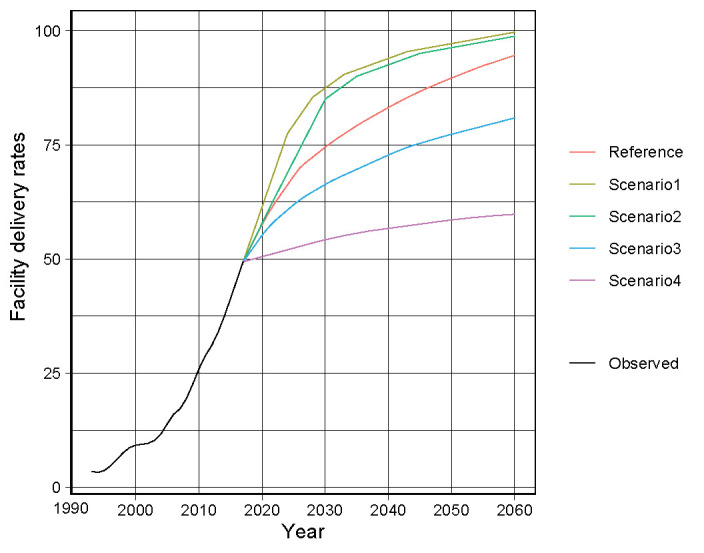
Facility delivery rates for the reference forecast and four scenarios.

The predicted values of the reference forecast and alternative scenarios from 2018 to 2060 are presented in **Figure 1**, **Table 2**, and Tables S2–S6 in the **Online Supplementary Document**. As mentioned earlier, Scenario 1 was the best scenario, Scenarios 2 and 3 were moderate scenarios, and Scenario 4 was the worst one. The parameters (*p*, *d*, and *q)* in the ARIMAX models given by the equation and the associated AIC are ARIMA (1,0,0) and 189.02, respectively. The coefficients and standard errors (SEs) are presented in **Table 3**.

**Table 2:** Predicted values of facility delivery rates for the reference forecast and four scenarios

Year	Reference (%)	Scenario 1 (%)	Scenario 2 (%)	Scenario 3 (%)	Scenario 4 (%)
2018	52.09	53.40	52.20	51.31	49.75
2019	54.81	57.40	55.00	53.25	50.13
2020	57.53	61.40	57.80	55.19	50.50
2021	60.00	65.40	60.60	56.88	50.88
2022	62.22	69.40	63.40	58.31	51.25
2023	64.19	73.40	66.10	59.50	51.63
2024	66.16	77.40	68.80	60.69	52.00
2025	68.13	79.40	71.50	61.88	52.38
2026	69.97	81.40	74.20	62.94	52.75
2027	71.19	83.40	76.90	63.88	53.13
2028	72.28	85.40	79.60	64.69	53.50
2029	73.38	86.40	82.30	65.50	53.88
2030	74.47	87.40	85.00	66.31	54.19
2031	75.50	88.40	86.00	67.06	54.50
2032	76.47	89.40	87.00	67.75	54.81
2033	77.38	90.40	88.00	68.38	55.13
2034	78.28	90.90	89.00	69.00	55.38
2035	79.19	91.40	90.00	69.63	55.63
2036	80.03	91.90	90.50	70.25	55.88
2037	80.81	92.40	91.00	70.88	56.13
2038	81.59	92.90	91.50	71.50	56.31
2039	82.38	93.40	92.00	72.13	56.50
2040	83.16	93.90	92.50	72.75	56.69
2041	83.91	94.40	93.00	73.34	56.88
2042	84.63	94.90	93.50	73.91	57.06
2043	85.31	95.40	94.00	74.44	57.25
2044	86.00	95.65	94.50	74.91	57.44
2045	86.69	95.90	95.00	75.31	57.63
2046	87.28	96.15	95.25	75.72	57.81
2047	87.88	96.40	95.50	76.13	58.00
2048	88.47	96.65	95.75	76.53	58.19
2049	89.05	96.90	96.00	76.92	58.38
2050	89.61	97.15	96.25	77.30	58.53
2051	90.15	97.40	96.50	77.65	58.69
2052	90.70	97.65	96.75	78.01	58.84
2053	91.24	97.90	97.00	78.37	59.00
2054	91.79	98.15	97.25	78.73	59.13
2055	92.30	98.40	97.50	79.08	59.25
2056	92.75	98.65	97.75	79.44	59.38
2057	93.20	98.90	98.00	79.80	59.50
2058	93.66	99.15	98.25	80.16	59.59
2059	94.11	99.40	98.50	80.51	59.69
2060	94.56	99.65	98.75	80.87	59.78

**Table 3:** Coefficient and standard error of parameters

Term	Coefficient	SE
AR	0.8836	0.0805
Intercept	957.3479	124.0487
SDI	−750.1663	376.5924
Distance to any health facility	−3.1196	0.8019
Quality ANC	3.2376	2.9765
Facility delivery	−3.6637	1.6866

ANC – antenatal care, AR – autoregressive, SE – standard error, SDI – socio-demographic index

**Figure 2:**
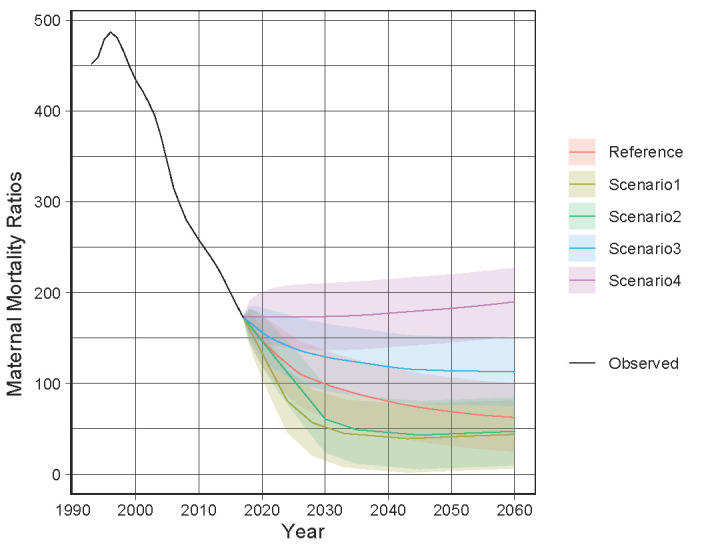
Observed and predicted maternal mortality ratios in Bangladesh from 1993 to 2060.

**Figure 2** shows the projected MMR for Bangladesh by scenario. The black lines are the previously observed values, and the coloured lines from 2018 are the predicted values. Scenario 1, the best-case scenario, had the lowest MMR, with a predicted MMR of below 70 per 100 000 live births in 2026. The predicted MMR in 2026 per 100 000 live births is 68.66 (95% PI = 33.389 to 103.93). Scenario 2, the moderate scenario, had the second-lowest MMR, with a predicted MMR of less than 70 per 100 000 live births in 2029. The predicted MMR of 69.40 per 100 000 live births is with a 95% PI = 33.04 to 105.78. The reference forecast had the third lowest MMR, with an MMR of 69.78 per 100 000 live births (95% PI = 32.44 to 107.11) in 2049. Although the MMR for Scenario 3 decreased slowly, it did not drop below 70 per 100 000 live births by 2060. Scenario 4, which had the highest MMR, also resulted in the MMR not falling below 70 per 100 000 live births until 2060.

For the evaluation of the model, the observed values of the MMR from 1993 to 2017 were fitted to the model created to visually evaluate the model (Figure S5 in the **Online Supplementary Document**).

## DISCUSSION

In this study, a reference forecast and four alternative scenarios were developed to predict when the MMR in Bangladesh would fall below 70 per 100 000 live births, which is the target of SDG 3.1. Scenario 1, the earliest and best of the scenarios, predicts an MMR of below 70 per 100 000 live births in 2026. Scenario 2 (moderate) predicts an MMR of below 70 per 100 000 live births in 2029, and Scenarios 1 and 2 would achieve SDG 3.1. The reference forecast would not achieve the target, but would have an MMR of below 70 per 100 000 live births in 2049. Scenarios 3 and 4 result in MMRs that would not be below 70 per 100 000 live births by 2060.

According to Kassebaum et al. (2014) [10], the 2030 MMR in Bangladesh would be 150–200 per 100 000 live births, making the results of this study optimistic. Kassebaum et al. (2014) [10] identified maternal deaths and estimated the MMR from 1900 to 2013 using a variety of data, including DHS (1993–1994, 1996–1997, 1999–2000, 2001); the estimated annualised rate of change from 2003 to 2013 was used to project the MMR for 2030. The description of the model for future projections was not detailed, but it was stated that a straightforward forecast scenario was created. In contrast, the present study used four variables to forecast future MMR beyond 2018, and these variables were selected based on a three delays model affecting maternal mortality. One variable, the institutional delivery rate, was used to create scenarios from 2018 to 2060. The data and methodology used to forecast the future MMRs differ from those used in the study by Kassebaum et al. (2014) [10], leading to differences in the results of the MMR forecasts.

The best model for this study was selected using AIC. Regarding the coefficients and SEs of the variables, the SE for the coefficient of the quality of ANC was very large (coefficient = 3.2376, SE = 2.9765), suggesting that the quality of ANC did not affect the outcome, namely, MMR, in the present model. Quality ANC was defined in this study as at least four ANC visits, with at least one weight and blood pressure measurement, urinalysis, and blood tests, and an explanation of the danger signs of complications [8]. The latest WHO guideline recommends an antenatal check-up schedule of at least eight contacts [20] because a decrease in the number of ANC visits has been shown to result in an increase in perinatal deaths [21]. In addition, maternal blood pressure measurements and proteinuria checks at each ANC visit are considered essential elements of good clinical practice [20]. The latest WHO guideline was published in 2016, and quality ANC as defined in the 2017–2018 DHS does not reflect the content of this guideline. The criteria for quality ANC are inadequate compared with international standards, and this may not have affected the MMR. The other variables were distance to any health facility (coefficient = −3.1196, SE = 0.8019), facility delivery (coefficient = −3.6637, SE = 1.6866), and SDI (coefficient = −750.1663, SE = 376.5924).

The variable of institutional delivery rate was used to create the scenarios. As mentioned earlier, Scenarios 1 and 2 meet the targets of SDG 3.1. For reasons, Scenarios 1 and 2 are ideal scenarios, in which the country's overall institutional delivery rate increases at a faster pace than in Scenarios 3 and 4, and both scenarios require an institutional delivery rate of at least 85% by 2030. Based on the percentage distribution of live births in the three years preceding the 2017–2018 DHS survey in Bangladesh, the number of live births in urban and rural areas was calculated using the number of live births in 2019, specifically, 774 376 in urban areas and 2 122 344 in rural areas [8, 22]. According to the health facility survey in 2017, there were 78 facilities in urban areas and 281 in rural areas that were equipped for normal deliveries [23]. Based on these available data, if 85% of the live births were institutional deliveries, each facility performs 8439 deliveries per year in urban areas and 6420 deliveries per year in rural areas. To simplify the calculations, the size of the health facility, the option of a cesarean section, and consideration of multiple births were not included in the calculations.

Similarly, using the latest institutional delivery rates of 62.5% (urban areas) and 44.6% (rural areas) [8], the number of institutional deliveries per facility was calculated as 6204 in urban areas and 3368 in rural areas. Based on the latest number of deliveries per facility, an 85% facility delivery rate would require 106 facilities in urban areas and 535 facilities in rural areas for normal deliveries, indicating a shortage of 28 and 254 facilities, respectively. In Bangladesh, the shortage of health facilities is already a major challenge, especially in rural areas, where the number of health facilities is inadequate and NGOs and private facilities are few. Therefore, the cooperation of the non-public sector is essential to increase institutional deliveries.

The reference forecast and Scenario 3 are scenarios that consider regional differences by administrative division. This is because several reports on Bangladesh have found that community and regional characteristics are associated with decisions regarding the place of birth and birth attendants [24, 25]. Bangladesh has eight administrative divisions, and the education level of women, wealth of households, and utilisation of maternal and child health services, such as ANC visits and facility delivery rates, vary by division. In particular, Sylhet, Mymensingh, and Barishal have higher percentages of women who have never received ANC services, high percentages of births by untrained traditional birth attendants, and low percentages of institutional deliveries [8].

The difference between Scenario 3 and the reference forecast was that Scenario 3 had lower annual increase rates for Sylhet, Mymensingh, Barishal, Chattogram, and Rangpur than the reference forecast. At this point, the reference forecast is considered the closest to the current situation. However, if the increase rate in areas with low utilisation of maternal and child health services, such as Sylhet, Mymensingh, and Barishal, is lower, the results would be similar to those of Scenario 3. Considering regional differences, a rapid increase in institutional deliveries only in the Dhaka, Khulna, and Rajshahi regions would not lead to a similar increase in institutional delivery rates for the country as a whole, and this is reflected in the MMR. Interventions that increase facility birth rates in areas with low facility birth rates are required to improve the MMR.

This study shows that an increase in the institutional delivery rate reduces the maternal mortality ratio. However, to increase the institutional delivery rate and contribute to the MMR, health care facilities must provide adequate quality care. A study analysing secondary data from Ghana showed that only the factor of delivering in a health facility did not decrease maternal mortality risks and that a high facility delivery rate did not necessarily lead to a reduction in maternal mortality [26]. However, the importance of giving birth in high-quality facilities is emphasised by the finding that intrapartum stillbirths were reduced only in the most capable facilities in Ghana [26]. The Janani Suraksha Yojana programme in India, the government programme of cash payments to women who give birth in health facilities to reduce maternal mortality, also increased the rate of institutional deliveries; however, no association was found between the Janani Suraksha Yojana programme and a reduction in maternal mortality [27]. Reasons for the lack of significant results include the low quality of care provided in health care facilities [26,27].

For health care facilities to provide quality health care, it is necessary to improve the quality and quantity of health care personnel and secure drugs, supplies, and medical equipment. Bangladesh has a shortage of medical human resources, with fewer nurses and midwives than doctors [28,29]. It is essential to train midwives, who play an important role in improving the health and saving the lives of mothers and newborns during pregnancy, childbirth, and the postpartum period [30]. According to United Nations Population Fund (UNFPA) et al. (2021) [22], Bangladesh had 4396 midwives in 2019, with 2 896 720 live births in the same year, and 0.3 midwives for every 10 000 people. Although an educational system for midwives has been developed, the number of midwives is inadequate considering this situation. The State of the World's Midwifery 2021 states that Bangladesh requires approximately 60 000 midwives [22]. This means that 3.7 midwives are needed for every 10 000 people. The WHO Global Strategic Directions for Nursing and Midwifery 2021–2025 identified four strategic directions: education of midwives and nurses, employment, service delivery, and leadership [31]. According to the UNFPA, no midwives assume leadership roles in the Ministry of Health [22]. Midwives need to take on a leadership role in midwifery education, which would improve their status, create a comfortable work environment, and ensure midwives are retained [31].

### Limitations of the study

This study had several limitations. Although there are various possible factors that can affect the MMR, the number of variables that could be included in the model equation was limited. Even variables that are associated with the MMR were excluded if they had few observed values during the target period, such as data related to postnatal care and Emergency obstetric and newborn care (EmONC). In addition, the scenarios in this study were designed to account for regional disparities; however, owing to data availability constraints and for simplicity of analysis, the only variable used was the facility delivery rate.

Bangladesh declared its first national lockdown on 26 March 2020 to control the spread of COVID-19, suspending road traffic and closing all non-essential organisations and educational institutions, leaving only medical services, pharmacies, and grocery stores open [32]. However, after the lockdown ended, its impact on the utilisation of medical services was confirmed. Another limitation of this study is that we did not consider this impact in calculating the percentage of women receiving quality ANC and institutional delivery rates, which were used as variables in the current study.

Additionally, most of the data used in the analysis were DHS data. Although the data were collected from respondents by trained personnel using a standardised questionnaire [13], some questions were related to past content, which may have led to recall bias.

The use of the ARMAX model also entails limitations. First, the model has a relatively large number of parameters, which may lead to unstable estimations and predictions, especially when the available time points are limited. Second, although it is a characteristic of predictive models, the accuracy of the ARMAX model tends to degrade when making long-term predictions.

## CONCLUSIONS

This study developed a reference forecast and four scenarios to predict when Bangladesh's MMR will fall below the SDG 3.1 target of 70 per 100 000 live births. The best scenario, Scenario 1, was the earliest, with an MMR of below 70 per 100 000 live births in 2026. Scenario 2, the forecast based on the national strategy for 2019–2030, predicted an MMR of below 70 per 100 000 live births in 2029, and these two scenarios would achieve SDG 3.1. The reference forecast would not achieve SDG 3.1, but it would have an MMR of <70 per 100 000 live births in 2049. There are regional differences in institutional delivery rates, and the reference forecast and Scenario 3 consider regional differences by administrative district. To increase the institutional delivery rate and reduce the MMR, as in Scenarios 1 and 2, it is necessary to improve the institutional delivery rate in regions with low institutional delivery rates. In addition, health facilities need to provide quality medical care to contribute to the reduction of the MMR by increasing the institutional delivery rate, as shown by the results of this study.

## Additional material


Online Supplementary Document
