# Supplementary material for: Projections of maternal mortality ratios in Bangladesh
Source: J Glob Health. 2024 Jan 26;14:04015. doi: 10.7189/jogh.14.04015 (PMC10811434; doi:10.7189/jogh.14.04015)
Supplement: Online Supplementary Document [file jogh-14-04015-s001.pdf]

Supplementary Material

Supplementary Tables

Table S1. GATHER Statement

Item #	Checklist item	Reported on page #
**Objectives and funding**		
**1**	Define the indicator(s), populations (including age, sex, and geographic entities), and time period(s) for which estimates were made.	3-8
**2**	List the funding sources for the work.	21
**Data Inputs**		
For all data inputs from multiple sources that are synthesized as part of the study:		
**3**	Describe how the data were identified and how the data were accessed.	3-4
**4**	Specify the inclusion and exclusion criteria. Identify all ad-hoc exclusions.	4-5
**5**	Provide information on all included data sources and their main characteristics. For each data source used, report reference information or contact name/institution, population represented, data collection method, year(s) of data collection, sex and age range, diagnostic criteria or measurement method, and sample size, as relevant.	3-4, 10
**6**	Identify and describe any categories of input data that have potentially important biases (e.g., based on characteristics listed in item 5).	NA
For data inputs that contribute to the analysis but were not synthesized as part of the study:		
**7**	Describe and give sources for any other data inputs.	NA
For all data inputs:		
**8**	Provide all data inputs in a file format from which data can be efficiently extracted (e.g., a spreadsheet rather than a PDF), including all relevant meta-data listed in item 5. For any data inputs that cannot be shared because of ethical or legal reasons, such as third-party ownership, provide a contact name or the name of the institution that retains the right to the data.	10-12
**Data analysis**		
**9**	Provide a conceptual overview of the data analysis method. A diagram may be helpful.	3
**10**	Provide a detailed description of all steps of the analysis, including mathematical formulae. This description should cover, as relevant, data cleaning, data pre-processing, data adjustments and weighting of data sources, and mathematical or statistical model(s).	3-8<br>Figure S1-4
**11**	Describe how candidate models were evaluated and how the final model(s) were selected.	7
**12**	Provide the results of an evaluation of model performance, if done, as well as the results of any relevant sensitivity analysis.	10<br>Figure S5
**13**	Describe methods for calculating uncertainty of the estimates. State which sources of uncertainty were, and were not, accounted for in the uncertainty analysis.	15-16
**14**	State how analytic or statistical source code used to generate estimates can be accessed.	21
**Results and Discussion**		
**15**	Provide published estimates in a file format from which data can be efficiently extracted.	10-11
**16**	Report a quantitative measure of the uncertainty of the estimates (e.g. uncertainty intervals).	Table S2-S6
**17**	Interpret results in light of existing evidence. If updating a previous set of estimates, describe the reasons for changes in estimates.	15-16
**18**	Discuss limitations of the estimates. Include a discussion of any modelling assumptions or data limitations that affect interpretation of the estimates.	19-20

Table S2. Observed and reference forecast of maternal mortality ratios in Bangladesh from 1993 to 2060

Year	Observed and reference forecast of maternal mortality ratios		
		95% prediction intervals	
1993	452.00	452.00	452.00
1994	459.00	459.00	459.00
1995	479.00	479.00	479.00
1996	486.94	486.94	486.94
1997	481.14	481.14	481.14
1998	466.87	466.87	466.87
1999	449.40	449.40	449.40
2000	434.00	434.00	434.00
2001	423.00	423.00	423.00
2002	410.00	410.00	410.00
2003	395.00	395.00	395.00
2004	372.00	372.00	372.00
2005	343.00	343.00	343.00
2006	315.00	315.00	315.00
2007	297.00	297.00	297.00
2008	280.00	280.00	280.00
2009	269.00	269.00	269.00
2010	258.00	258.00	258.00
2011	248.00	248.00	248.00
2012	238.00	238.00	238.00
2013	227.00	227.00	227.00
2014	214.00	214.00	214.00
2015	200.00	200.00	200.00
2016	186.00	186.00	186.00
2017	173.00	173.00	173.00
2018	164.63	147.15	182.12
2019	156.16	132.82	179.49
2020	147.66	120.63	174.69

2021	140.06	110.46	169.66
2022	133.36	101.90	164.83
2023	127.57	94.72	160.41
2024	121.75	87.87	155.63
2025	115.93	81.26	150.59
2026	110.54	75.27	145.81
2027	107.44	71.70	143.17
2028	104.78	68.68	140.87
2029	102.11	65.74	138.48
2030	99.43	62.84	136.01
2031	96.96	60.21	133.72
2032	94.72	57.84	131.60
2033	92.70	55.71	129.68
2034	90.66	53.60	127.73
2035	88.62	51.50	125.75
2036	86.80	49.63	123.98
2037	85.21	48.00	122.42
2038	83.60	46.36	120.84
2039	81.99	44.73	119.25
2040	80.38	43.10	117.66
2041	78.87	41.57	116.16
2042	77.47	40.16	114.77
2043	76.18	38.87	113.49
2044	74.88	37.56	112.20
2045	73.58	36.26	110.91
2046	72.62	35.30	109.95
2047	71.66	34.33	108.99
2048	70.69	33.36	108.02
2049	69.78	32.44	107.11
2050	68.92	31.58	106.26
2051	68.12	30.78	105.45
2052	67.31	29.97	104.65
2053	66.50	29.17	103.84

2054	65.69	28.35	103.03
2055	65.00	27.66	102.34
2056	64.52	27.18	101.86
2057	64.05	26.71	101.39
2058	63.57	26.23	100.91
2059	63.09	25.75	100.43
2060	62.61	25.27	99.95

Maternal mortality ratios are expressed per 100,000 live births.

Table S3. Observed and predicted maternal mortality ratios for scenario 1

Year	Observed and predicted maternal mortality ratios for scenario 1.		
		95% prediction intervals	
1993	452.00	452.00	452.00
1994	459.00	459.00	459.00
1995	479.00	479.00	479.00
1996	486.94	486.94	486.94
1997	481.14	481.14	481.14
1998	466.87	466.87	466.87
1999	449.40	449.40	449.40
2000	434.00	434.00	434.00
2001	423.00	423.00	423.00
2002	410.00	410.00	410.00
2003	395.00	395.00	395.00
2004	372.00	372.00	372.00
2005	343.00	343.00	343.00
2006	315.00	315.00	315.00
2007	297.00	297.00	297.00
2008	280.00	280.00	280.00
2009	269.00	269.00	269.00
2010	258.00	258.00	258.00
2011	248.00	248.00	248.00
2012	238.00	238.00	238.00
2013	227.00	227.00	227.00
2014	214.00	214.00	214.00
2015	200.00	200.00	200.00
2016	186.00	186.00	186.00
2017	173.00	173.00	173.00
2018	159.85	142.36	177.33
2019	146.68	123.34	170.01
2020	133.49	106.45	160.52
2021	120.28	90.68	149.88

2022	107.05	75.59	138.52
2023	93.81	60.97	126.66
2024	80.56	46.68	114.44
2025	74.62	39.95	109.28
2026	68.66	33.39	103.93
2027	62.69	26.96	98.43
2028	56.71	20.62	92.81
2029	54.39	18.02	90.76
2030	52.05	15.46	88.63
2031	49.70	12.95	86.45
2032	47.34	10.46	84.23
2033	44.98	7.99	81.96
2034	44.43	7.37	81.50
2035	43.88	6.76	81.00
2036	43.32	6.15	80.49
2037	42.75	5.54	79.96
2038	42.18	4.94	79.42
2039	41.60	4.34	78.86
2040	41.01	3.73	78.29
2041	40.42	3.13	77.71
2042	39.82	2.52	77.13
2043	39.22	1.91	76.53
2044	39.53	2.21	76.85
2045	39.83	2.51	77.16
2046	40.13	2.80	77.46
2047	40.42	3.09	77.76
2048	40.72	3.38	78.05
2049	41.00	3.67	78.34
2050	41.28	3.95	78.62
2051	41.56	4.23	78.90
2052	41.84	4.50	79.18
2053	42.11	4.77	79.45
2054	42.38	5.04	79.72

2055	42.65	5.31	79.99
2056	42.92	5.58	80.26
2057	43.18	5.84	80.52
2058	43.44	6.10	80.78
2059	43.70	6.36	81.04
2060	43.95	6.61	81.29

Maternal mortality ratios are expressed per 100,000 live births.

Table S4. Observed and predicted maternal mortality ratios for scenario 2

Year	Observed and predicted maternal mortality ratios for scenario 2		
		95% prediction intervals	
1993	452.00	452.00	452.00
1994	459.00	459.00	459.00
1995	479.00	479.00	479.00
1996	486.94	486.94	486.94
1997	481.14	481.14	481.14
1998	466.87	466.87	466.87
1999	449.40	449.40	449.40
2000	434.00	434.00	434.00
2001	423.00	423.00	423.00
2002	410.00	410.00	410.00
2003	395.00	395.00	395.00
2004	372.00	372.00	372.00
2005	343.00	343.00	343.00
2006	315.00	315.00	315.00
2007	297.00	297.00	297.00
2008	280.00	280.00	280.00
2009	269.00	269.00	269.00
2010	258.00	258.00	258.00
2011	248.00	248.00	248.00
2012	238.00	238.00	238.00
2013	227.00	227.00	227.00
2014	214.00	214.00	214.00
2015	200.00	200.00	200.00
2016	186.00	186.00	186.00
2017	173.00	173.00	173.00
2018	164.24	146.76	181.73
2019	155.47	132.14	178.80
2020	146.68	119.64	173.71
2021	137.86	108.26	167.47

2022	129.04	97.57	160.50
2023	120.56	87.72	153.40
2024	112.07	78.19	145.95
2025	103.56	68.89	138.23
2026	95.04	59.77	130.31
2027	86.51	50.77	122.24
2028	77.96	41.87	114.06
2029	69.41	33.04	105.78
2030	60.84	24.26	97.43
2031	58.49	21.74	95.25
2032	56.14	19.25	93.02
2033	53.77	16.78	90.75
2034	51.39	14.33	88.46
2035	49.01	11.89	86.13
2036	48.45	11.28	85.62
2037	47.88	10.67	85.09
2038	47.31	10.07	84.55
2039	46.73	9.47	83.99
2040	46.14	8.86	83.42
2041	45.55	8.26	82.84
2042	44.95	7.65	82.26
2043	44.35	7.04	81.66
2044	43.74	6.42	81.06
2045	43.13	5.81	80.45
2046	43.43	6.10	80.76
2047	43.72	6.39	81.05
2048	44.01	6.68	81.35
2049	44.30	6.96	81.63
2050	44.58	7.25	81.92
2051	44.86	7.52	82.20
2052	45.14	7.80	82.48
2053	45.41	8.07	82.75
2054	45.68	8.34	83.02

2055	45.95	8.61	83.29
2056	46.21	8.87	83.55
2057	46.48	9.14	83.82
2058	46.74	9.39	84.08
2059	46.99	9.65	84.34
2060	47.25	9.91	84.59

Maternal mortality ratios are expressed per 100,000 live births.

Table S5. Observed and predicted maternal mortality ratios for scenario 3

Year	Observed and predicted maternal mortality ratios for scenario 3		
		95% prediction intervals	
1993	452.00	452.00	452.00
1994	459.00	459.00	459.00
1995	479.00	479.00	479.00
1996	486.94	486.94	486.94
1997	481.14	481.14	481.14
1998	466.87	466.87	466.87
1999	449.40	449.40	449.40
2000	434.00	434.00	434.00
2001	423.00	423.00	423.00
2002	410.00	410.00	410.00
2003	395.00	395.00	395.00
2004	372.00	372.00	372.00
2005	343.00	343.00	343.00
2006	315.00	315.00	315.00
2007	297.00	297.00	297.00
2008	280.00	280.00	280.00
2009	269.00	269.00	269.00
2010	258.00	258.00	258.00
2011	248.00	248.00	248.00
2012	238.00	238.00	238.00
2013	227.00	227.00	227.00
2014	214.00	214.00	214.00
2015	200.00	200.00	200.00
2016	186.00	186.00	186.00
2017	173.00	173.00	173.00
2018	167.50	150.01	184.98
2019	161.88	138.55	185.21
2020	156.25	129.22	183.28
2021	151.51	121.91	181.11

2022	147.68	116.21	179.14
2023	144.74	111.90	177.58
2024	141.79	107.91	175.67
2025	138.82	104.16	173.49
2026	136.30	101.03	171.57
2027	134.23	98.49	169.96
2028	132.60	96.50	168.69
2029	130.96	94.59	167.33
2030	129.31	92.72	165.89
2031	127.88	91.12	164.63
2032	126.66	89.78	163.55
2033	125.67	88.68	162.65
2034	124.67	87.60	161.73
2035	123.66	86.53	160.78
2036	122.64	85.47	159.81
2037	121.62	84.41	158.83
2038	120.58	83.35	157.82
2039	119.55	82.28	156.81
2040	118.50	81.22	155.78
2041	117.57	80.27	154.86
2042	116.74	79.44	154.04
2043	116.02	78.71	153.33
2044	115.53	78.21	152.85
2045	115.26	77.93	152.58
2046	114.98	77.66	152.31
2047	114.71	77.38	152.04
2048	114.42	77.09	151.76
2049	114.20	76.86	151.53
2050	114.03	76.69	151.36
2051	113.91	76.58	151.25
2052	113.80	76.46	151.14
2053	113.68	76.34	151.02
2054	113.55	76.21	150.89

2055	113.43	76.09	150.77
2056	113.30	75.96	150.64
2057	113.17	75.82	150.51
2058	113.03	75.69	150.37
2059	112.90	75.55	150.24
2060	112.76	75.42	150.10

Maternal mortality ratios are expressed per 100,000 live births.

Table S6. Observed and predicted maternal mortality ratios for scenario 4

Year	Observed and predicted maternal mortality ratios for scenario 4		
		95% prediction intervals	
1993	452.00	452.00	452.00
1994	459.00	459.00	459.00
1995	479.00	479.00	479.00
1996	486.94	486.94	486.94
1997	481.14	481.14	481.14
1998	466.87	466.87	466.87
1999	449.40	449.40	449.40
2000	434.00	434.00	434.00
2001	423.00	423.00	423.00
2002	410.00	410.00	410.00
2003	395.00	395.00	395.00
2004	372.00	372.00	372.00
2005	343.00	343.00	343.00
2006	315.00	315.00	315.00
2007	297.00	297.00	297.00
2008	280.00	280.00	280.00
2009	269.00	269.00	269.00
2010	258.00	258.00	258.00
2011	248.00	248.00	248.00
2012	238.00	238.00	238.00
2013	227.00	227.00	227.00
2014	214.00	214.00	214.00
2015	200.00	200.00	200.00
2016	186.00	186.00	186.00
2017	173.00	173.00	173.00
2018	173.22	155.74	190.71
2019	173.33	150.00	196.66
2020	173.42	146.39	200.45
2021	173.49	143.89	203.10

2022	173.55	142.09	205.01
2023	173.59	140.75	206.43
2024	173.62	139.74	207.50
2025	173.63	138.96	208.30
2026	173.63	138.36	208.90
2027	173.61	137.88	209.35
2028	173.59	137.49	209.68
2029	173.55	137.18	209.92
2030	173.73	137.14	210.32
2031	173.90	137.15	210.65
2032	174.06	137.18	210.95
2033	174.21	137.23	211.20
2034	174.59	137.52	211.65
2035	174.95	137.83	212.07
2036	175.31	138.13	212.48
2037	175.66	138.45	212.87
2038	176.23	138.99	213.47
2039	176.79	139.53	214.05
2040	177.35	140.07	214.63
2041	177.90	140.61	215.20
2042	178.45	141.15	215.75
2043	178.99	141.68	216.30
2044	179.53	142.21	216.85
2045	180.06	142.74	217.38
2046	180.59	143.26	217.92
2047	181.11	143.78	218.44
2048	181.63	144.30	218.96
2049	182.15	144.81	219.48
2050	182.77	145.44	220.11
2051	183.40	146.06	220.73
2052	184.02	146.68	221.36
2053	184.63	147.29	221.97
2054	185.36	148.02	222.70

2055	186.09	148.75	223.43
2056	186.81	149.47	224.15
2057	187.53	150.19	224.87
2058	188.36	151.02	225.70
2059	189.19	151.85	226.53
2060	190.03	152.68	227.37

Maternal mortality ratios are expressed per 100,000 live births.

Supplementary Figures

Figure S1. Interpolation of maternal mortality ratios.

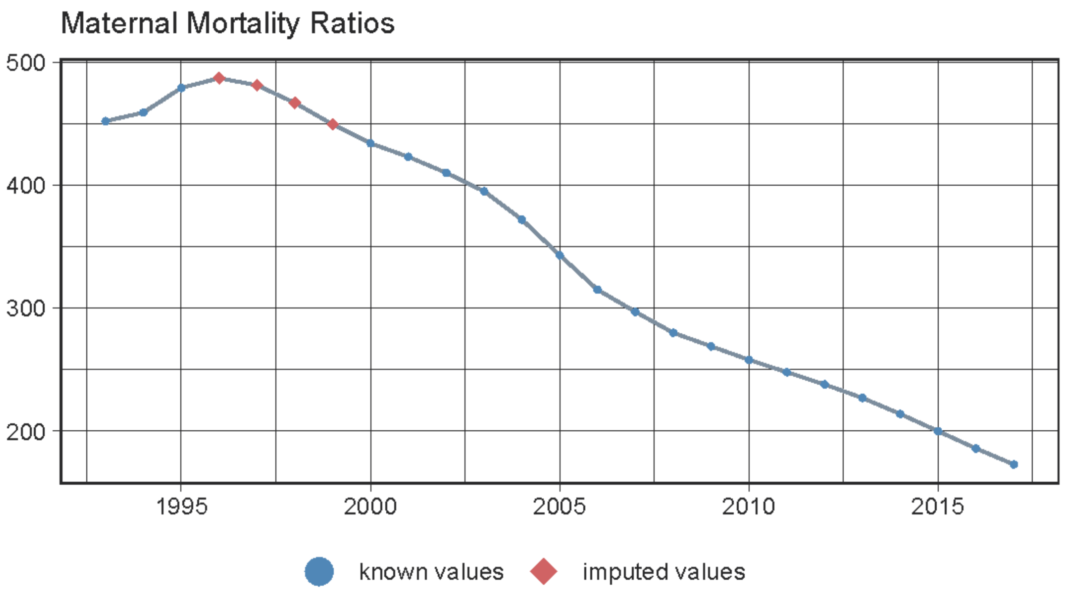

Maternal mortality ratios are expressed per 100,000 live births.

Figure S2. Interpolation of percentage of sample clusters with any health facilities within 5 km.

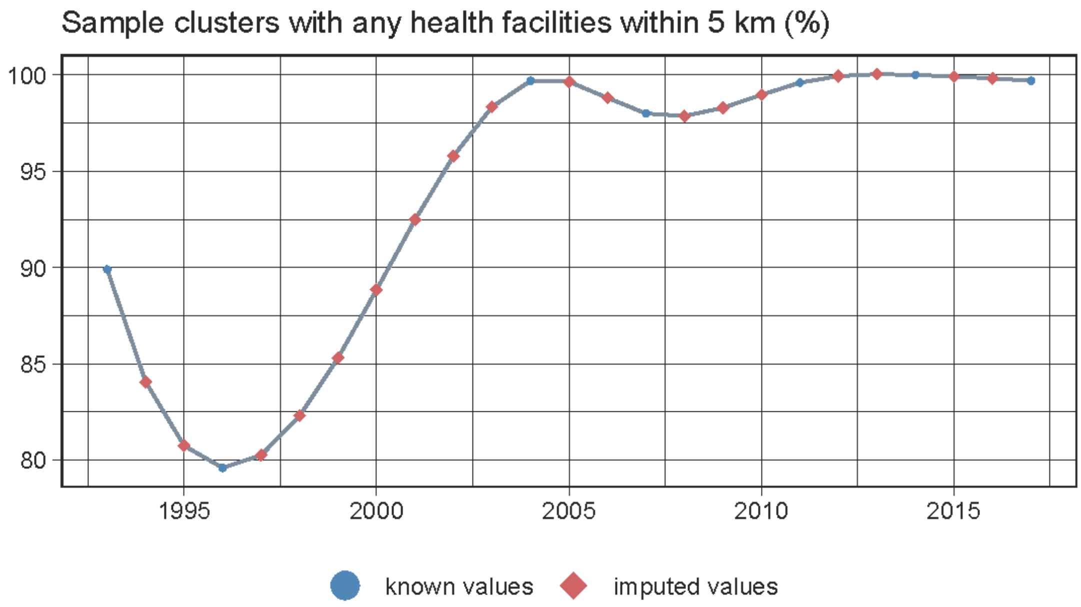

Figure S3. Interpolation of Percentage of Women Receiving Quality ANC.

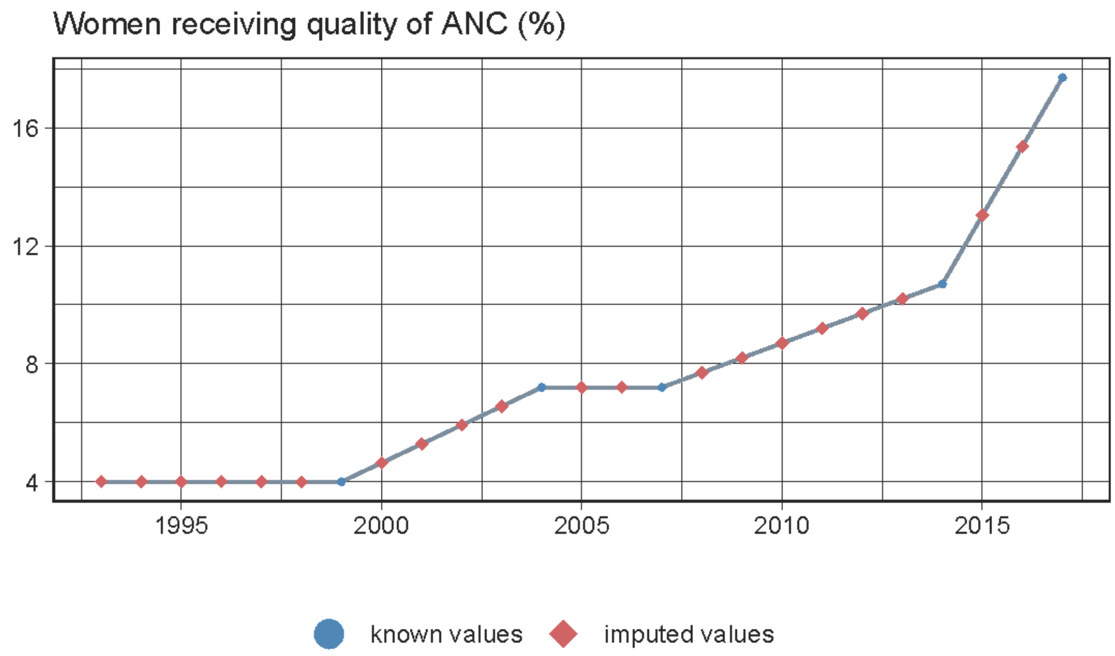

Figure S4. Interpolation of facility delivery rates.

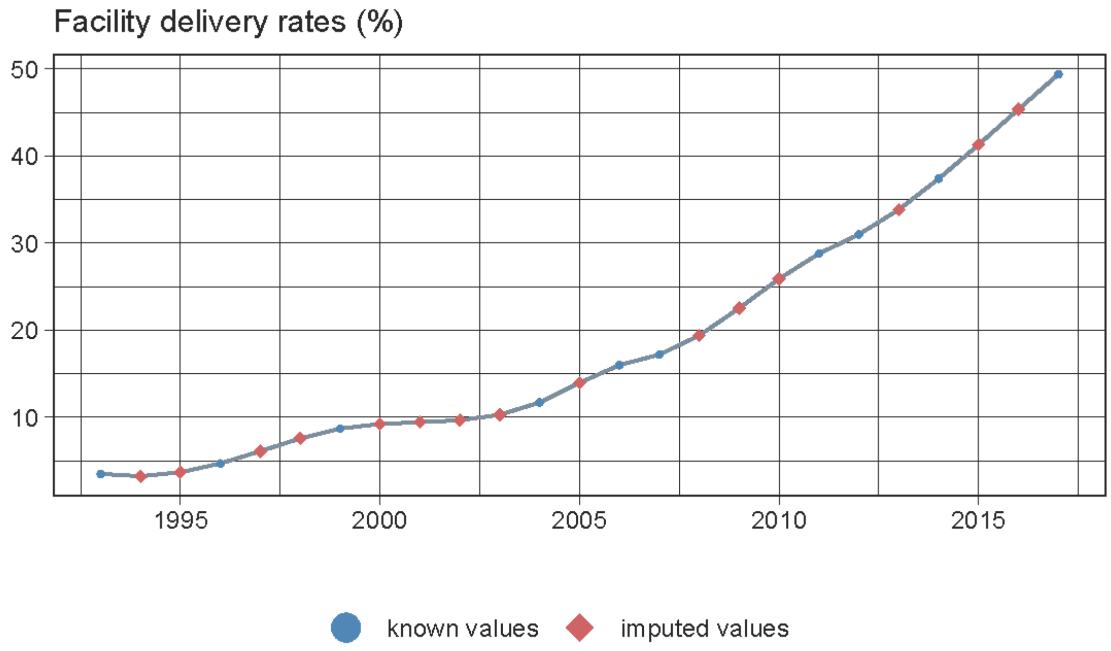

Figure S5. Observed and predicted maternal mortality ratios from 1993 to 2017.

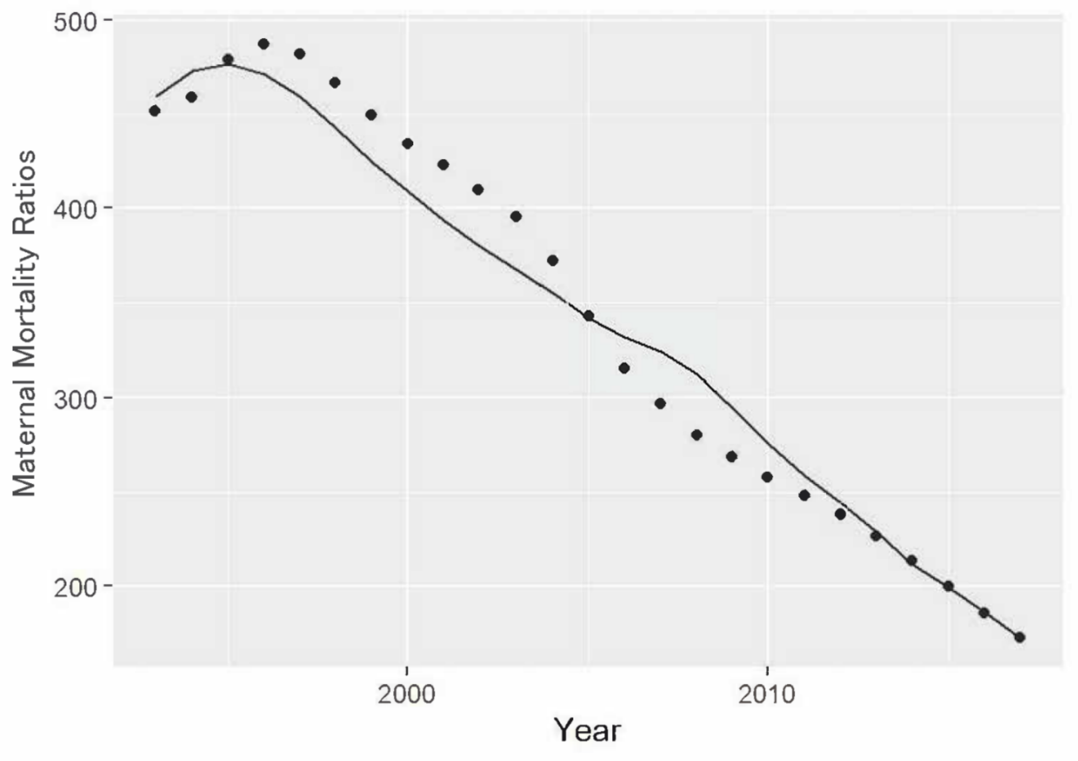

Maternal mortality ratios are expressed per 100,000 live births.

Line: predicted maternal mortality ratios, Points: observed maternal mortality ratios.
